# Effectiveness of health education as an intervention designed to prevent female genital mutilation/cutting (FGM/C): a systematic review

**DOI:** 10.1186/s12978-018-0503-x

**Published:** 2018-04-12

**Authors:** Susan Waigwa, Lucy Doos, Caroline Bradbury-Jones, Julie Taylor

**Affiliations:** 1 School of Nursing, College of Medical and Dental Science, University of Birmingham, Edgbaston, Birmingham, B15 2TT UK; 2 School of Social policy, College of Social Sciences, University of Birmingham, Birmingham, UK; 3 School of Nursing, College of Medical and Dental Sciences, University of Birmingham and Birmingham Women’s and Children’s Hospital NHS Foundation Trust, Birmingham, UK

**Keywords:** Community, ‘Circumcision, Female’, Prevention, Female genital mutilation, Health education

## Abstract

**Background:**

Female Genital Mutilation/Cutting (FGM/C) is a harmful practice that violates the human rights of women and girls. Despite global efforts to restrict the practice, there have been few reports on major positive changes to the problem. Health education interventions have been successful in preventing various health conditions and promoting service use. They have also been regarded as promising interventions for preventing FGM/C. The objective of this systematic review is to synthesise findings of studies about effectiveness of health education as an intervention to prevent FGM/C.

**Methods:**

The electronic databases searched were MEDLINE, EMBASE, Cochrane library, Web of Science, Psych INFO, CINAHL and ASSIA. Our search included papers published in the English language without date limits. Study quality was assessed using the Mixed Methods Appraisal Tool (MMAT). A predesigned data recording form was used to extract data from the included studies which were summarised by comparing similar themes.

**Results:**

Twelve out of 359 individual studies met our inclusion criteria. Seven studies were quantitative, three were qualitative and two used mixed methods. Six studies tested before and after the interventions, four studies assessed the effectiveness of previous interventions used by different research teams and two studies endorsed the intervention. Four main factors emerged and were associated with facilitating or hindering the effectiveness of health education interventions: sociodemographic factors; socioeconomic factors; traditions and beliefs; and intervention strategy, structure and delivery.

**Conclusions:**

It is vital to target factors associated with facilitating or hindering the effectiveness of health education for FGM/C. This increases the possibility of effective, collective change in behaviour and attitude which leads to the sustainable prevention of FGM/C and ultimately the improved reproductive health and well-being of individuals and communities.

**Electronic supplementary material:**

The online version of this article (10.1186/s12978-018-0503-x) contains supplementary material, which is available to authorized users.

## Plain English summary

Female genital mutilation (FGM/C) is a harmful practice that involves total or partial removal of female genitalia without medical purpose. It is mainly practised in some countries in Africa, Asia, the Middle East and some communities in South America. Migration, however, has been associated with the wide spread of FGM/C around the globe. It is performed on young girls and causes short-term and life–long consequences for women as well as extended consequences for families and the community at large. These consequences increase burden to the health systems. Health education interventions are among the prominent forms of interventions that can prevent the practice of FGM/C. However, its impact is dependent on factors that facilitate or hinder effectiveness. Our review revealed that these factors include sociodemographic factors; socioeconomic factors; traditions and beliefs; and intervention strategy, structure and delivery. To ensure the effectiveness of health education interventions, these factors should be considered.

In conclusion, health education interventions have the potential to prevent FGM/C. They can produce a sustainable impact on the reproductive health and well-being of individuals as well as communities. The findings from this study imply that, with caution, health education interventions that focus on FGM/C can be effectively implemented in different populations.

## Background

Female genital mutilation/cutting (FGM/C) is a violation against the human rights of women and children such as the right to freedom from discrimination, torture and violence; the right to health; and the right to education. FGM/C involves the total or partial removal of female genitalia without medical purpose. The global prevalence of FGM/C among girls and women is estimated to be over 200 million. It is concentrated particularly in some African, Asian and Middle Eastern countries [1–3]. However, migration has been associated with the wide spread of FGM/C around the globe [4]. Records from 2012 estimate that about 513,000 girls and women had either undergone or were at risk of FGM/C in the United States of America (USA) [5, 6]. In 2015, England and Wales, in the United Kingdom (UK), recorded 137,000 girls and women subjected to FGM/C and 60,000 girls at risk [7].

The World Health Organization (WHO) classifies FGM/C into four types; Type I- Clitoridectomy; Type II- Excision; Type III- Infibulation; and Type IV- Other procedures, including piercing and incising. The practice has been associated with adverse short-term health consequences such as heavy bleeding and tetanus infections; and long-term consequences such as recurring vaginal and pelvic infections, menstrual complications, difficulties during pregnancy and childbirth; and psychological problems such as Post-Traumatic Stress Disorder (PTSD), anxiety and depression [8–11].

FGM/C, which is usually performed on young girls between the age of infancy and 15 years [12], has no medical benefits and medical professionals around the globe are prohibited from carrying out the practice [4, 13]. Whilst the Universal Declaration of Human Rights as well as other global conventions and declarations emphatically oppose the practice [14]. There are a number of factors that allow FGM/C to continue. These include cultural/traditional factors, which are tied up with rituals and complex belief systems [15], religious factors, which are enforced by specific religious beliefs and teachings [16], and health/hygiene factors, which include myths associated with perceived health benefits [17].

The attempts to deal with the negative consequences of FGM/C have unfortunately developed into the medicalisation of the procedure, whereby guarantees of safety are erroneously proffered as a reason for FGM/C to be carried out by health professionals. However, the engagement of health professionals in such procedures inevitably cause harm and constitutes a violation of medical conduct [4, 13, 18]. Consequently, greater effort has been made to deter health professionals from engaging in FGM/C by legal consequences of the practice. However, despite the global efforts to curb FGM/C, there has been few reports on major positive changes of the problem [1].

Health education is the main intervention of interest in this review. It involves different learning experiences designed to help individuals and communities improve their health by increasing their knowledge or influencing their attitude [19]. This goes beyond sharing or disseminating information about a health issue to address motivation, skills, confidence, and communication of information. Differences in economic, social and environmental conditions; individual risk factors and behaviours; and use of health systems are also considered [20].

It is vital for health education interventions to aim at long-term changes to the health behaviour and the norms that are attributed to a health problem. However, evaluation of the effectiveness of interventions depend on documenting the outcomes, effects, formation, process, cost-effectiveness and benefits of the interventions [20].

Health education programmes have been effective in addressing various health related issues such as smoking uptake and cessation, healthy pregnancy and improved newborn outcomes [21–23]. Health education has also succeded in promoting the use of services such as family planning, particularly in communities that are reluctant to access such services [24]. It has also been considered to be a potential intervention for preventing FGM/C. There are some studies which have reported successful health education interventions in preventing FGM/C globally, but there is need for more exploration of the interventions including their effects in different communities [25]. To our knowledge, there is no systematic review that has synthesised the evidence and ensured understanding of the effectiveness of health education interventions as discrete interventions for FGM/C. The purpose of this review therefore, was to explore the effectiveness of health education as an intervention to prevent FGM/C in the affected communities.

## Methods

We searched electronic databases for published work using comprehensive search strategies. Seven main international databases were systematically searched. These included; MEDLINE, EMBASE, Cochrane library, Web of Science, Psych INFO, CINAH and ASSIA. These databases were selected to best represent source material in health, applied health, and human science. Grey literature was also searched and the reference lists from included studies and systematic reviews about FGM/C interventions were hand searched. Search terms were structured carefully to include the problem, intervention comparator and expected outcomes (using a PICO formulation). The terms included female genital mutilation OR female circumcis* OR female genital cutting, affected communit*, health educat* AND/OR health literacy, prevent* OR abandon* OR eliminat* OR stop*(see Additional file 1). English articles with no date restriction were searched. The search was completed in June 2016. Endnote × 7 was utilised as the main reference manager.

The first author (SW) screened titles independently and a second reviewer (HS) independently repeated the process to ensure no relevant studies were excluded. The same reviewers independently decided on the full texts to be included by scrutinising the abstracts. Predetermined inclusion and exclusion criteria were used to guide the screening and selection process.

We included studies focusing on communities affected by FGM/C. There was no limit to the population by geographical location. The included studies either used or discussed health education as an intervention. They had a purpose of disseminating information to individuals or groups of people with an aim of preventing FGM/C as the primary outcome. We considered all study designs with no defined publication timeframe. We excluded studies that did not focus on communities affected by FGM/C and those that focused on medical or cosmetic procedures like vulvectomy or labiaplasty. Studies with a focus on other interventions and studies involving circumcision/genital cutting other than female genital cutting, for instance male circumcision were also excluded.

Data from included studies were extracted using a predesigned data recording form, including general details of the study, intervention description, study outcomes and conclusions. Data were recorded on Microsoft Excel software, which the team used to crosscheck extraction details and ensure accuracy. Discrepancies were discussed and agreed upon within the review team.

Study quality was assessed using the Mixed Methods Appraisal Tool (MMAT) Version 2011 [26]. This was chosen because of its ability to review mixed method studies alongside qualitative and quantitative studies in a single combined tool.

The included papers did not have data that were suitable for meta-analysis, similar to Yang et al. [27], due to heterogeneity. We therefore carried out a thematic analysis focusing on the main themes that were evident in the included studies. Both manifest and latent themes were explored and described as understood by the authors.

## Results

**Fig. 1:**
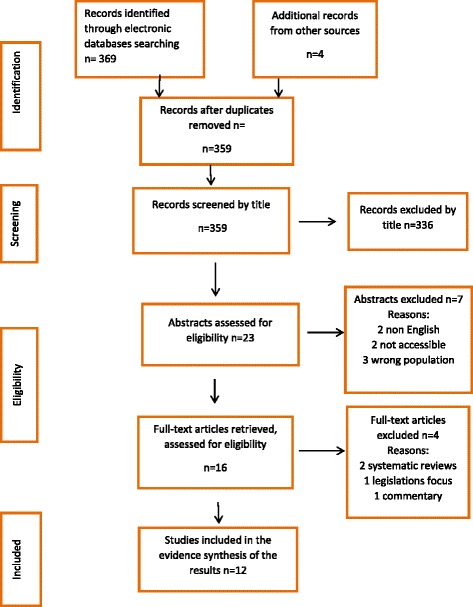
PRISMA Flow chart

The search elicited 359 publications from which 12 full text articles met our inclusion criteria, as shown in the PRISMA flow chart (Figure 1).

The populations involved in the 12 included studies were from Africa and the Middle East, where FGM/C is prevalent. Half of the studies (six) tested knowledge about FGM/C before and after intervention [28, 29, 30, 31, 32, 33]. Four different studies assessed the effectiveness of previous interventions used by different research teams [34, 35, 36, 37]. The remaining two studies endorsed the intervention as a solution for preventing FGM/C following evidence of the relevance of health education interventions to communities affected by FGM/C [38, 39].

From the 12 included studies, seven were quantitative, three qualitative and two used mixed methods. The quality mean-score, of all included studies was 83.33%, which was above the predetermined cut-off mark of 50%, as measured with the Mixed Method Assessment Tool (MMAT). All of the included studies scored above 50% in each of their respective categories and therefore qualified for inclusion. Table 1 presents a detailed summary of each of the 12 included studies.

**Table 1:** Summary of included papers

Effectiveness of health education as an intervention designed to prevent female genital mutilation/cutting (FGM/C): A systematic review.							
Author and year	Setting and prevalence	Population	Sample type and recruitment strategy	Study design and comparison group	Method and quality of studies	Information/activities intervention offered/evaluated	Outcome/results
Ajuwon J Ademola, Brieger R William, Oladepo Oladimeji, deniyi D Joshua (1995)	South West Nigeria FGM/C Type I	Male practitioners Males and females community leaders Males and females in focus groups Nigeria, Yoruba community	Male and female community leaders Married and unmarried men and women Practitioners/ circumcisers Leaders in community Community members Practitioners age 45 and 70 years Community leaders and focus groups, not specified	Qualitative	Interviews 75%	General knowledge about FGM/C	There was high need for health education interventions especially for indigenous surgeries
Allam MF, Irala-Esteves DJ, Navajas FCR, Castillo DSA, Hoashi JS, Pankovich MB, Liceaga RJ. (2001)	Universities in Cairo, Egypt	Males and females Egypt	University students mean age of 20.97 years of medical and 20.73 years from non-medical Belong to a community that practice FGM	Cross-sectional No comparison group	Face-to-face interviews 32-item questionnaire 100%	General information about FGM	High proportion considered discussions in the media to play an important role in banning of FGM/C People are aware of the dangers involved, are more likely to be against the practice
Alo & Gbadebo, (2011)	Southwest Nigeria	Women Southwest Nigeria	Women who have at least one living daughter Belong to a community affected by FGM/C 15–49 years	Survey	Interviews 50%	General knowledge about FGM/C	Respondents with post-secondary education were at least likely to have their daughters cut. Only 52% of the women were aware of the health hazards associated with FGC Participants from high socio-economic status are least likely to have their daughters cut
Asekun-Olarinmoye EO,Amusan OA (2008)	Shao community is in Kwara State Nigeria Between 60 and 70% FGM/C Type I and II	Males and females Yoruba, Nigeria	Residents of Shao town above 10 years Participants belonged to a community that practice FGM/C Modal age of 30–39 and 20–29 in pre-intervention and post intervention respectively	survey	Questionnaires 100%	Health talks in vernacular on female genital anatomy, nature and types of FGM/C,complications, beliefs that encourage it Pictures were utilised to illustrate female genitalia, different types of mutilation Questions and answer sessions utilised for further discussions	No statistical significance difference between the composition and socio-demographic characteristics Education status, age and gender were found to be statistically significant in association to those who had their daughters excised Positive impact of the health education intervention on the attitude of the respondents to FGM/C and intentions to subject their daughters
Awuah JB (2008)	Aboabo No.1 - Suburb of Kumasi 75–85% prevalence (24.5% of women) FGM/C Type II	Females African-Ghana	Those whose address contacts could be traced to their homes Participants belong to a community that practice FGM No indicated age	Exploratory research	Face to face interviews 75%	Background, knowledge and experiences of being circumcised and facing birth complications participant Suggestions of ways to prevent FGM/C from the participants	43% suggested health professionals should use health talks 14% suggested use of dramas and role plays by health educators 14% believe that education of females would help 4% thought use of mass media to educate the public would help
Babalola S,Brasington A, Agbasimalo A, Helland A, Nwanguma E, Onah N. (2006)	Enugu state: 3 local government areas; i. Uzo-Uwani,ii. Isi-Uzo and iii. Enugu South FGC prevalence of Enugu −59% Ebonyi −78% Usually type I and II of FGC are performed	Males and females Africa-Enugu and Ebonyi states, Nigeria	Participants belong to communities affected by FGC a. Enugu state for intervention b.Ebonyi state for comparison c. age 18 to 59 d. men and women	cross-sectional survey Ebonyi state for comparison	Interviews 100%	Examination of knowledge, attitudes and practices surrounding women’s reproductive health Support and training in development of action plan Discussions on social and health complications of FGC	Increased knowledge leading to widespread intentions not to practice Increased self-efficacy to refuse pressure to perform Extremely religious people are less likely to abandon FGC Large number of children was associated with intentions not to practice
Chege J, Askew I, Igras S, Mutesh JK. (2004)	Semi-arid rural in Ethiopia and Kenya Ethiopia-Awash Woredea. Kenya-Ifo in Dadaab 76% (Ethiopia) 34% (Kenya) Specific community: 91% (Ethiopia) 100% (Kenya) FGM/C Type III	Males and females African-Ethiopians and Somali in Kenya	Participants must have experienced or lived with people who have experienced FGM Ethiopia-8 to 60 years Kenya-15 to 60 years Participants belong to communities affected by FGC	Quasi-experimental Ethiopia-six villages in Amibara Woreda. Kenya-Hagadera camp	Interviews 75%	Community level education outreach activities using behaviour-communication-change Community level advocacy Training dispensary service providers in treating complications and counselling clients on FGC related areas	Percentage of those who support abandonment in Ethiopia intervention group increased by 32%-control group increased by 10%-Kenya-intervention group remained at 23%-comparison group increased by 8% Percentage of those who do not intend to cut –Ethiopia intervention group increased by 26%-control reduced by 1%-Kenya intervention group increased by 3%-comparison increased by 8% Lower levels of exposure to FGC information translates to lower increases in positive attitudes and intent behaviours.
Diop NJ, Askew I (2009	Kolda Region in Southern Senegal 94% prevalence FGM/C Type I and II	Men and women Senegal	Males and females from villages where TOSTAN programme had been implemented and in Older than 15 years	Survey Quasi-experimental, pre-and post-intervention longitudinal design Comparison- villages where the programme had not reached	Interviews 100%	Modules about: Human rights, Problem-solving process, Basic hygiene and Women’s health	Statistically significant differences in the proportion of girls reported to have been cut in intervention group Significant attitudinal and behavioural changes leading to mass declaration against FGM/C Education, facilitated rapid change in traditional behaviours
Jacoby SD, Lucarelli M, Musse F, Krishnamurthy A, Salyers V (2015)	Lewiston, Maine United States. FGM/C Type I – IV	Somali Women, Individuals who had experienced perinatal health care	Somali women Living in Lewiston, Maine Participants were from countries where FGM/C is practiced 12 to 60 years	Mixed-methods	Interviews 75%	General information about women’s health including FGM/C	No participant had adequate health literacy Historietas were unanimously approved As appropriate health education tools
Mounir G, Nehad HM, Ibtsam MF. (2003)	Alexandria University, Egypt	Female students Egypt-Middle East	Students from Alexandria University second grade Participants belong to community affected by FGM Mean-19.35	Quasi-experiment El-Shatby hostel was the control group that did not receive the program	Questionnaire 75%	Training on Importance of premarital counselling, family planning, breastfeeding, sexually transmitted diseases Alternative methods of family planning, weaning and importance of breastfeeding, importance of antenatal care, methods of prevention of STDs Experience and precautions against FGM and early marriage, social pressure on early marriage and FGM	Statistically significant improvement in each domain of knowledge measured in intervention group and no absolute change was detected in the control group 33.3% gain scores was detected for knowledge about the term RH and FGM In regards to effects of intervention program, those of high social class had a higher post-test score The program resulted to significant improvement in most of knowledge items and a shift towards a positive attitude
Olaitan LO (2010)	3 State Capitals in South west Nigeria	Males and females African-Nigeria (Yoruba, Fulani, Hausa and Nupe)	Parents Participants belong to communities affected by FGC 15 to 65 and above	Survey No comparison group	Questionnaire 75%	General knowledge about FGM	No significance difference existed between males and females in the knowledge about FGM/C There was significant difference based on age in knowledge about FGM/C There was significant difference based on educational status Community health education is the best means of providing health information and education to people at every level.
Ruiz JI, Martinez AP, Bravo PMDM. (2015)	Spain-Murcia and Eastern Morocco	Males African-Living in Spain and	Male, living in Spain and Morocco originally from countries where FGM is performed Participants lived at least 18 years in their countries of origin and have personally being in contact with women with FGM Participants Comprehend Spanish or French Between 20 and 53 years	Qualitative	Semi-structured interview 75%	First-hand knowledge of the practice and its foundations-from various sensitisation and personal experience	Sensitised men can change viewpoints regarding the practice Important to use visual and communication media in health education programmes There is need for new development of health education programmes.

### Factors affecting the effectiveness of health education

Four major themes were identified: sociodemographic factors; socioeconomic factors; traditions and beliefs; and intervention strategy, structure and delivery. These are described further in this section in a nonlinear process. All the studies highlighted at least two themes and discussed their contribution in either enabling or hindering the effectiveness of health education intervention. A summary of the themes is presented in Table 2.

**Table 2:** Summary of themes in each study

Articles\themes	Sociodemographic						Socioeconomic		Traditions and beliefs			Intervention strategy, structure and delivery					
	Age	Ethnicity	Language	Gender	Marital status	Residential status	Education	Occupation/Role in community	Religion	Prevalence rate of community	location	Programme approach	Workshops	Counseling	Media	Graphics/artistic	Campaigns
Ajuwan et al. (1995)					✘											✘	
Allam et al. (2001)							✘		✘			✘			✘		
Alo & Gbadebo (2011)	✘						✘	✘		✘	✘						
Asekun-Olainmoye & Amusan (2008)							✘	✘	✘	✘							
Awuah (2008)			✘		✘					✘							
Babalola et al. (2006)		✘		✘								✘	✘		✘		
Chege et al. (2004)		✘			✘	✘		✘	✘		✘	✘			✘		
Diop and Askew (2009)		✘		✘	✘							✘	✘		✘		✘
Jacoby et al. (2015)			✘			✘								✘		✘	
Mounir et al. (2003)					✘		✘					✘					
Olaitano (2010)	✘						✘										
Ruiz et al. (2015)											✘					✘	✘

### Sociodemographic factors

Six sociodemographic elements were described in the included studies. They include age, ethnicity, language, gender, marital status and residential status.

#### Age

Two of the twelve studies reported that the age of the populations involved in health education interventions influenced the effectiveness of the interventions. In general, younger populations were more amenable to the interventions. In a study by Olaitan [36] with parents in Nigeria, knowledge of older parents was found to be significantly different from that of younger parents. In the same vein, Alo and Gbadebo [39] concluded that among populations that approved the practice, the levels of FGM/C prevalence were higher among older respondents. They suggested this was because younger respondents were more likely to be school educated, which increased their chances of engaging with FGM/C health education that encouraged abandonment of the practice.

#### Ethnicity

Three studies reported that ethnic differences between facilitators and communities sometimes influenced the effectiveness of health education interventions due to backlash. It was concluded that facilitators and interviewers needed to belong to the same ethnic groups as participants. Partnering with communities prevented a top-down approach that enhanced the effectiveness of the interventions [28, 30, 31]. If facilitators and interviewers were of different ethnicity from that of the participants, they were required to familiarise themselves with the cultural and structural customs by integrating with the communities beforehand [28, 31].

#### Language

Two studies highlighted the potential of language barriers reducing the effectiveness of health education interventions [32, 35]. Facilitators struggled with delivering messages and participants also found it hard to understand the messages which were not in their local language. Jacoby et al. found that the use of a ‘cultural broker’, who is a translator, helped in mitigating some of the language challenges by translating for both the facilitators and participants in languages they could understand [32].

#### Gender

Two studies reported that gender differences of the recipients affected levels of participating in health education interventions [28, 31]. For example, in the study by Babalola et al. [28], the measure of programme exposure where the radio was the major source revealed that 67.1% of men were exposed to at least one component of the programme, from which they learnt more about FGM/C, compared to 61.4% of women. Diop and Askew [31] in their report on evaluating the effectiveness of education offered by the ‘Tostan programme’ in Senegal, revealed that there were gender differences in awareness of at least two consequences of FGM/C. Among men, awareness increased from 11% to 80% and among women, from 7% to 83%. The slight difference between the genders was attributed to women’s personal experiences of undergoing FGM/C which facilitated a better understanding of the topics.

#### Marital status

Three studies reported that some married participants, both males and females, did not benefit from health education interventions. This is because they held on to their belief that FGM/C is a means of controlling promiscuity of girls and women which was a virtue in these communities [31, 35, 38]. However, although FGM/C traditionally was believed to be a prerequisite for marriage, some studies reported that most unmarried participants did not think it was important and they appreciated the health education interventions. The authors concluded that this reflected a natural decline in the practice due to generational differences [30, 33]. Chege et al. [30] on a different note, did not find significant association between marital status and support for or opposition of FGM/C and therefore concluded that being married did not act as a barrier to effective health education interventions.

#### Residential status

The residential status of intervention groups influenced the effectiveness of health education interventions, especially for participants who were immigrants. Jacoby et al. [32] indicated that refugees in the USA shared common concerns in health care matters. These included general health literacy levels and knowledge of the health implications of FGM/C. Additionally, Chege et al. [30] reported that there were higher chances of encountering more resistance to the intervention from immigrants due to forced law enforcement against FGM/C in the host country.

### Socioeconomic factors

Two socioeconomic factors emerged from the included studies; education and occupation/roles in communities.

#### Education

Five studies reflected on the extent to which levels of basic education impacted on access to and acceptability of FGM/C health education programmes. Asekun-Olarinmoye and Amusan [29] reported that education levels of the participants determined the acceptability and effectiveness of health education. This manifested in the attitudes expressed by those with no formal education who were more likely to encourage FGM/C. Olaitan [36] similarly reported that there was a significant difference in knowledge about FGM/C based on educational status. Those with more years of education had greater knowledge influenced by health education interventions. Alo and Gbadebo [39] also highlighted that parents with post-secondary education were less inclined to have their daughters undergo FGM/C because they were more likely to be exposed to health education interventions about the practice. They reported that 48% of those with post-secondary education had none of their daughters cut, compared to 20% of respondents with no formal education. Mounir et al. [33] reported that, students from higher income families had better improvement in knowledge about FGM/C because their family educational background supported acquisition of such knowledge. Slightly contradictorily, Allam et al. [34] found a considerable amount of ignorance concerning FGM/C existed among the educated population in Egypt, including some doctors and midwives.

#### Occupation/roles in communities

The studies by Asekun-Olarinmoye and Amusan [29] and Alo and Gbadebo [39] highlighted that traditional excisors, health professionals, community leaders and religious leaders were not only recipients of health education interventions, but also implementers and change agents for better outcomes of interventions. When such influential individuals refrain from supporting anti-FGM/C messages, it can negatively affect the success of health education interventions[30].

### Traditions and beliefs

#### Religion

One study concluded that religious belief was not associated with encouraging the continuation of FGM/C and therefore, not a hindrance to effective health education intervention [29]. However, Allam et al. [34] and Chege et al. [30] reported that religious affiliations of either the participants or the facilitators of health education interventions were likely to affect the effectiveness of the intervention. Distrust predominantly arose when programme facilitators affiliated themselves with a different religion from that of the participants. They also found that involving religious leaders in the health education programmes either positively influenced communities through their teachings about FGM/C, which encouraged abandonment of the practice, or negatively promoted the practice by for instance, referring to it as a religious requirement. Allam et al. [34] emphasised that it was more difficult to educate participants who believed FGM/C is a religious requirement for any faith, because they were more likely to condone the practice.

#### Prevalence of FGM/C in communities

Prevalence rates were presented to have an effect on health education interventions. For example, in the study from Ghana by Awuah [35], 100% (*n* = 70) of the respondents claimed that FGM/C was practiced, an exercise of which about 43% did not regret. Asekun-Olainmoye and Amusan [29] similarly indicated that 88% (*n* = 211) of female respondents reported to have had FGM/C, of whom 85% had no regrets. Alo and Gbadebo [39] reported on differences in prevalence of FGM/C between generations. They observed that attitude and prevalence more often than not go together. This was reflected in the insignificant 4% intergenerational difference that indicated minimal changes in attitudes following health education interventions that did not justify the efforts made to prevent the practice.

#### Locality

Three studies demonstrated the impact that locality has on the effectiveness of the intervention [30, 39]. Chege et al. [30], found that the percentage exposed to anti-FGM/C messages in Ethiopia, among the intervention group, increased from 21% to 71% while in Kenya, it increased from 40% to 59%. The difference was attributed to disparities in societal structures. For example, laws against FGM/C were harsher in Kenya, and their enforcement made it harder for interventions to win trust from FGM/C affected communities. Alo and Gbadebo [39] emphasised that, females in a rural setting were more likely to support FGM/C compared to those living in urbanised communities. They concluded that health education interventions in the rural areas require more intense planning and implementation than the urban areas. Ruiz et al. [37] in the same vein reported that awareness efforts in rural areas needed to be more intense in comparison with urban areas because the inherent isolation in rural areas propagated FGM/C.

### Programme strategy, structure and delivery

#### Programme approach

Methods that health education programmes used to approach the intervention groups were associated with the effectiveness of the interventions. Chege et al. [30], Mounir et al. [33] and Allam et al. [34] reported that it was necessary for health education facilitators to approach communities with caution; otherwise, they would face rejection. In studies that included interviewing as a process of health education, interventions were more effective when males interviewed males and females interviewed females [34]. Diop and Askew noted that researchers needed to integrate with community interviewers, who belonged to the target communities, prior to implementation so as to increase reliability of respondents reports [31].

Four studies showed that the levels of programme exposure impacted on the effectiveness of the health education intervention [28, 30, 31, 34]. Lower levels of exposure translated to a smaller increase in positive attitude and intended behaviour [30], while higher levels of exposure translated to powerful, effective means for facilitating rapid communal changes [31].

#### Workshops

The studies by Babalola et al. [28] and Diop and Askew [31] reported that there was inconsistency in lectures and workshops attendance. This influenced the effectiveness of the health education interventions as it translated to suboptimal outcomes because they yielded insignificant results.

#### Counselling

Jacoby et al. [32] showed that counselling early in the antepartum period was more effective than late counselling. This was preferable to the participants on the basis that early intervention gives ample time for thinking and discussing health concerns with spouses.

#### Media

Media seemed to be a vital tool for delivering FGM/C health education interventions. Radio appeared to be an effective means to reach the men in most populations and was mostly favourable among young people [28, 31, 34]. However, in the study by Chege et al. [30], media was disadvantageous because some messages were not always in support of FGM/C prevention. This in turn limited the effectiveness of the health education intervention.

#### Graphics/artistic

The use of graphics or artistic modes of dissemination mostly enhanced the effectiveness of health education interventions. Jacoby et al. [32] in their study about immigrant Somali women’s health literacy and perinatal experiences found that Historietas (graphical booklets) were endorsed by participants because they understood the contents better [32]. Practical training was reported by participants as a preference and was endorsed as a means of effective communication of anti-FGM/C including to traditional excisors [37, 38].

## Discussion

This review aimed to assess the effectiveness of health education interventions in preventing FGM/C in the affected communities. We managed to unveil factors that facilitate or hinder effectiveness of health education interventions. Various studies, including systematic reviews, have evaluated different interventions as well as their benefits and effectiveness in preventing FGM/C. Health education, among other interventions, has been regarded as important in contributing to raising awareness about FGM/C, leading to changed attitudes and behaviours in various communities [40, 41].

Our study challenges the approach applied by previous reviews about the effectiveness of FGM/C interventions in general. We ventured to explore health education as an individual intervention, while focusing on the issues that are specific to this particular intervention. Our results show that the effectiveness of FGM/C health education interventions depended on factors linked to sociodemographic factors; socioeconomic factors; traditions and beliefs; and intervention strategy, structure and delivery. The most pronounced finding was that these factors are guaranteed to disturb the process of implementing change through health education interventions. It is therefore, important to ensure that health education interventions have tailored information, communication and education to fit the target population based on their needs. This requires prior understanding of individual capacity and existing knowledge including individual ages and levels of education [29, 30, 33, 35, 36, 37, 38, 39]. The importance of community-based approaches for FGM/C health education interventions cannot be underestimated. The value of this approach is demonstrated by Chege et al. [30] who reported how religious leaders and other key leaders in the communities were used for advocacy against FGM/C. Community leaders are valued individuals and their inclusion in interventions has been recognised to enhance effectiveness particularly where messages relate to sensitive health problems such as HIV [42]. There are a number of strategies to support the process. For example, Mounir et al. [33] described how intervention facilitators in their study dressed in a similar style of clothing to participants in an attempt to encourage shared identity and break down barriers.

It is important to acknowledge that the studies included in this review focused on different communities who may have varying reasons for performing FGM/C, even when they come from the same country. This supports the importance of tailoring interventions to the target population and minimising generalisation.

Personal beliefs and views have been highlighted in a number of contexts to affect attempts to induce change of negative social behaviours because they are highly influenced by prior knowledge, experience and psychological state [43, 44]. An understanding of individual viewpoints and attitude can predict behaviour change [45]. This review has shown that educational background, rate of prevalence of FGM/C, religion and media all influence the effectiveness of health education interventions [28, 29, 30, 31, 34, 35, 39]. Diop and Askew [31], for example contended that there was a reduction in the number of daughters who were cut after their mothers participated in a programme aimed at changing perceptions of FGM/C. Alo [39], however, reported that women’s decisions did not matter as their husbands had control over decision-making, influencing their behaviour. This indicated that though the women were aware of the health issues, FGM/C would still be practised. Michie et al. [46] similarly suggested that behaviour change is dependent on psychological capabilities such as strength and skill that could affect individual perception and social opportunities such as cultural norms that could influence behaviour, regardless of individual perception.

While changed attitude and behaviour by individuals is essential, sharing acquired information and change is as important for better results of collective prevention of FGM/C [30]. The willingness to share information however, is dependent on factors such as commitment, enjoyment of helping others, reputation and organisational reward [47]. Diop and Askew [31] for example, indicated that women who participated in an FGM/C programme were encouraged to “adopt” a friend/relative and share information regarding their learning during classes and this proved effective. The programme encouraged sharing of information by establishing community management committees to strengthen village ownership of the programme. However, it is not always a guarantee that group prevention is attained, especially if pro-FGM/C messages are shared instead of messages against FGM/C. When correct information is communicated and shared effectively, it eventually results in collective knowledge and awareness that in turn influences communal change. This can culminate in wider results for public campaigns and denouncement of the practice [30].

This review acknowledges the factor of acculturation, which has the capacity to influence the attitude and views of immigrant communities, depending on the economic status and legislative changes of the home or host countries [48]. Two studies that were conducted in Spain and USA focused on communities with a history of FGM/C living in these high income countries. They found that participants’ views and opinions may not be entirely free from acculturation [32, 37]. In addition, the impact of acculturation may not be reflected differently when the host country is a low-middle income country [30] .

According to the WHO, health education presents to communities a package comprising opportunities for learning that are based on sound theories to offer health information. From some perspectives, the FGM/C interventions can be perceived as a top-down approach, with communities being the recipients[19]. With this in mind, Babalola et al. (28) emphasises the importance of integration with communities prior to implementation of interventions. They argued that this increases community acceptance of an intervention, leading to its success.

Findings from this review highlight health education as a promising intervention in preventing FGM/C. The intervention is favoured over other interventions such as legislation, because it is less repressive. Although the law reduces the rate of FGM/C, it has also been found to drive the practice underground. In other instances, the law has led to parents subjecting girls to FGM/C at a younger age before they are susceptible to anti-FGM/C messages. There is also an association between law enforcement and increased medicalisation as well as reduced reporting of FGM/C cases [37, 49–54].

Contextualising health education interventions is only possible when there is sufficient consideration of the characteristics of target populations. Contextualising involves inclusion of the communities in planning the programmes, for instance, involving permanent residents who belong to the target communities as facilitators or research assistants [29]. Religious and other key leaders can also help to promote the interventions [30]. Community members can be involved in dissemination of information to relatives and friends, therefore, encouraging public awareness and resistance to FGM/C [31]. It is evident from this review that tailoring information to fit the needs of the target populations is crucial because it increases the acceptability of the programme and influences quicker dissemination of information among communities.

Despite the strengths, this review was limited because some studies did not indicate the duration of the interventions. It is therefore important to note that based on the nature of the intervention, shorter durations may have offered less chance for programmes to attain desired goals, especially in sharing of information. This review also considered only studies reported in English. Other languages could have reported the issue differently for instance, studies from non-English speaking high-income countries. The review considered only studies that focused on affected communities and excluded studies from non-practicing communities which future research should consider to include.

There is ample room to improve women and girls’ safety from FGM/C. Rational approaches through health education interventions should be carefully planned. As Abdulcadir et al. [55] points out, there is a dearth of research focusing on interventions to prevent FGM/C. This includes health education offered by health professionals who work with communities affected by FGM/C. Further research is needed to establish the effectiveness of health education interventions offered to different populations living in high-income countries. Cultural competency especially in the healthcare system can help improve health outcomes and quality of care [56]. Further research therefore is needed to increase the understanding of how best to involve different demographic groups including non-practicing communities, in health education interventions, in order to maximise effective prevention of FGM/C.

## Conclusion

Health education is an important intervention which has the capacity to change deeply engraved beliefs and attitudes attributed to certain health problems such as FGM/C. When the intervention is comprehensively planned, implemented and evaluated, it can be successful in preventing FGM/C in any target group. This study contributes to the understanding of the facilitators and barriers of effective health education interventions in preventing FGM/C. Our findings suggest that health education interventions have the potential to influence communal change, which eventually leads to sustainable prevention of FGM/C. The success of health education interventions is dependent on sociodemographic elements, socioeconomic factors, traditions and beliefs and programme approach. Evidence suggests that these factors are vital and require intensive consideration at every stage of the intervention. This ensures increased possibility of influencing communal change in behaviour and attitude, leading to sustainable prevention of FGM/C, thus, improved reproductive health and wellbeing of individuals and communities.

## Additional file

Additional file 1: Search strategies. (DOCX 12 kb)

## Abbreviations

CBJ: Caroline Bradbury-Jones
FGM/C: Female genital mutilation/cutting
HS: Harpreet Sihre
JT: Julie Taylor
LD: Lucy Doos
SW: Susan Waigwa
UK: United Kingdom
USA: United States of America
WHO: World Health Organization
