# Supplementary material for: Effectiveness of health education as an intervention designed to prevent female genital mutilation/cutting (FGM/C): a systematic review
Source: Reprod Health. 2018 Apr 12;15:62. doi: 10.1186/s12978-018-0503-x (PMC5897952; doi:10.1186/s12978-018-0503-x)
Supplement: Supplementary file 1 — Search strategies. (DOCX 12 kb) [file 12978_2018_503_MOESM1_ESM.docx]

**Additional File 1: Search strategies**

**MEDLINE search strategy database: Ovid MEDLINE**

**Number of hits: 38**

1. Circumcision, Female/

2. ((Female adj5 ('genital mutilation' or circumcis* or 'genital cutting')) or FGM* or FGC).mp.

3. Health Education/

4. (health adj5 (educat* adj3 communit*)).mp.

5. health literacy.mp. or Health Literacy/

6. (communit* adj5 (practic* or affect*)).mp.

7. (prevent* or aboli* or abandon* or eliminat* or stop*).mp.

8. 1 or 2

9. 4 or 5

10. 3 or 9

11. 6 and 7 and 8 and 10

**Web of science search strategy: Webofknowledge.com**

**Number of hits: 169**

#1 (female genital mutilation)

#2 (female near5 'genital mutilation' or circumcis* or 'genital cutting')

#3 (health education)

#4 ((health near5 'educat* or communit*'))

#5 (health literacy)

#6 (prevent* or aboli* or abandon* or eliminat* or stop*)

#7 #2 OR #1

#8 #5 OR #4 OR #3

#9 #8 AND #7 AND #6

**Cochrane Library search strategy: Wiley.com**

**Number of hits: 6**

#1 female genital mutilation

#2 female adj5 ((genital mutilation) or circumcis* or (genital Cutting))

#3 health education

#4 health adj5 (educat* adj3 (communit*))

#5 health literacy

#6 MeSH descriptor: [Health Literacy] explode all trees

#7 MeSH descriptor: [Health Education] explode all trees

#8 MeSH descriptor: [Circumcision, Female] explode all trees

#9 prevent* or aboli* or abandon* or eliminat* or stop*

#10 #1or #2 or #8

#11 #4 or #5 or #6

#12 #3 or #7

#13 #11 or #12#

14 #9and #10 and #13
